# Heuristic rules for cooperative transport in wood ant nest maintenance

**DOI:** 10.1242/jeb.252597

**Published:** 2026-07-23

**Authors:** Natalie Arscott, Cornelia Buehlmann, Andrew Philippides, Paul Graham

**Affiliations:** School of Life Sciences/School of Engineering and Informatics, University of Sussex, Brighton, BN1 9QG, UK

**Keywords:** Self-organisation, Collective behaviour, Collaborative transport, Wood ants, Collective construction

## Abstract

Collective behaviour in ants includes cooperative transport where individuals control behaviour relative to an object. Some species show efficient collective food transport because individuals share directional information. It is less clear how individuals coordinate in tasks, such as movement of nest material, where movement direction is unclear. Wood ants build nests from twigs and leaf litter that require constant maintenance. We provoked such maintenance by placing sticks over nest entrances which ants removed by walking backwards aligned to the stick. When ants cooperated, the second ant joined a moving stick more quickly and precisely than initial ants. Cooperation was further promoted by ants staying engaged for longer and making fewer grip adjustments when cooperation was effective. Although coordination was stochastic and imprecise, ants respond to local sensory feedback, with adjustments that promote cooperation. This suggests collective transport can emerge from simple and flexible sensorimotor heuristics.

## Teaser

**Summary:** Cooperative behaviour during nest maintenance in wood ants emerges from simple and flexible sensorimotor heuristics.

## INTRODUCTION

Social insects demonstrate remarkable collective behaviours and provide powerful systems for understanding how complex group-level organisation can emerge from simple local interactions. Construction, decision making and foraging organisation can emerge from self-organising principles (Camazine et al., 2001; Czaczkes et al., 2015; Feinerman and Korman, 2017; Gordon, 2019; Visscher, 2007), where global pattern and order emerge from interactions among lower-level components of the system. In social insect colonies, individuals follow behavioural rules utilising local information available to them, which emergently leads to collective behaviour (Camazine et al., 2001; Fewell, 2015).

One fascinating large-scale collective behaviour is nest construction, where colonies of social insects create and maintain large, complex structures without top-down organisation (Franks et al., 1992; Guimaraes et al., 2018; Khuong et al., 2016; Trocki et al., 2026; Tschinkel, 2004, 2005). Nest construction also involves smaller scale collective behaviours whereby groups of workers move pieces of nest material together (Moffett, 1987). This so-called cooperative transport (Berman et al., 2011; Czaczkes and Ratnieks, 2013; McCreery and Breed, 2014; Moffett, 1992) is a cooperative behaviour that benefits individuals (West et al., 2007) by enabling a task to be performed more efficiently. While individuals gain short-term advantages by sharing the load, it ultimately supports overall colony performance. Cooperative transport, defined as two or more individuals moving an item simultaneously, is common in ants and has been recorded in at least 40 genera (Hölldobler and Wilson, 1990; Moffett, 1992; Moffett, 2010). It typically involves transporting food to the nest (Cerdá et al., 2009; Chatterjee et al., 2025; Czaczkes et al., 2011; Traniello, 1983). In contrast, cooperative transport during nest construction or maintenance is poorly understood, despite occasional reports of workers moving nest material (Moffett, 1987) or brood (Moffett, 1992).

Collaborative transport occurs on a continuum from highly organised with coordination rules and task separation, through to poorly organised behaviours where multiple individuals try to move an object together in an uncoordinated way (reviewed in Czaczkes and Ratnieks, 2013; McCreery and Breed, 2014), resulting in deadlocks and movement inefficiencies (Chauvin, 1950; Moffett, 1992; Sudd, 1965).

In highly organised cooperative transport, ants show cooperation skills via adapted behaviours not observed during individual transport. For instance, *Pheidole crassinoda* workers usually lift and carry small objects forwards but drag large objects backwards. However, during cooperative transport, they walk backwards dragging an object when they are at the front but lift and carry it walking forward when they are at the back (Sudd, 1960). Such adapted walking patterns are key for well-coordinated transport, but there are also changes in how and where ants grasp an object when in a group. Some ants grasp an item with their mandibles when solitarily carrying it but push with their head and legs when moving cooperatively (Moffett, 1988). Furthermore, ants grasp objects in non-random ways, i.e. often grasping them at the front, back or at a corner, avoiding the sides when carrying solitarily but not when in groups (Czaczkes et al., 2011; Czaczkes and Ratnieks, 2013; Sudd, 1965).

A central challenge in collective behaviour is understanding how individuals coordinate their actions when they have no access to global information. Coordination can occur via physical forces applied to the object, which act as a form of communication (Czaczkes and Ratnieks, 2011; Dreyer et al., 2025; Feinerman et al., 2018; Gelblum et al., 2015). When an individual ant alters its direction, speed or position, these changes generate subtle mechanical cues such as shifts in tension, compression or torque that propagate through the object. Other ants detect these cues and adjust their behaviour accordingly, enabling coordinated movement without centralised control or explicit communication.

Wood ants build large dome-shaped nests in woodlands using twigs, pine needles, leaves or other organic material (Chauvin, 1959a; Kristiansen and Amelung, 2001; Wisniewski, 1967). Mounds with several hundred-thousand workers (Hölldobler and Wilson, 1990) can reach up to 2 m in height, though are typically less than 1 m high (Frouz et al., 2016; Hölldobler, 1960). The complex nest architecture incorporates tunnels and passages for thermoregulation (Horstmann and Schmid, 1986; Kadochová and Frouz, 2014), living chambers and a subterranean section (Goryunov, 2015). The mound's loose, malleable material allows it to be easily reconfigured in response to environmental changes; therefore, workers continuously move nest material during maintenance behaviours (Chauvin, 1958, 1959a,b; Coenen-Stass et al., 1980; Goryunov, 2015).

We leveraged this maintenance behaviour to investigate the mechanisms of cooperative transport, by asking how individuals and pairs of ants move sticks that are blocking a nest entrance. How do wood ants achieve coordinated movement of an object when individuals lack a shared directional goal? Specifically, we asked whether the second ant joining a stick modifies its behaviour in ways that facilitate the movement of the stick.

## MATERIALS AND METHODS

### Ants and experimental setup

Data were collected in July and August 2019, in the laboratory and in natural woodland. Wood ants (*Formica rufa* Linnaeus 1761) build large nest mounds that can house up to 100 queens and between 100,000 and 400,000 workers without forming distinct castes (Collingwood, 1979). Colony fragments of several thousand ants were collected at Broadstone Warren, East Sussex, UK, and kept in the laboratory in large containers (1.25 m×0.65 m×0.6 m) at constant temperature of 25–27°C. Ants were fed *ad libitum* with sucrose, dead crickets and water. Field experiments were performed in Abbot's Wood, East Sussex, UK.

**Fig. 1.**
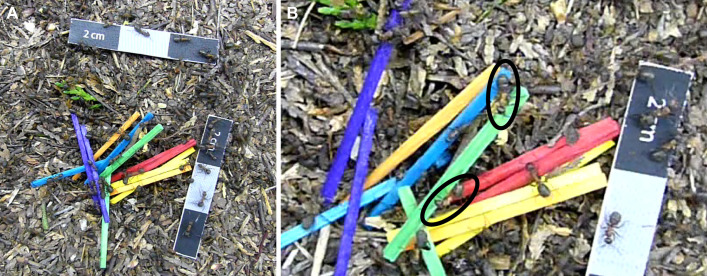
**Sticks covering a wood ant nest entrance.** (A) Coloured sticks placed over a nest entrance. (B) Two ants (ringed) move a stick together.

For the behavioural recordings, 5–24 pieces of coloured sticks (2–5 cm in length) were placed over a nest entrance at assorted orientations (Fig. 1). Because nests typically have many entrances, we could place the sticks over different entrances throughout the experiment. We varied both the number and length of sticks to mimic the natural heterogeneity of physical obstructions. As soon as the sticks were placed down, the nest entrance was filmed from a distance of about 30 cm for 30 min, using a Panasonic DMC-FZ200 camera. Recordings were done at four different nests on multiple days from 11:00 h to 16:00 h BST.

### Video analysis

From a set of 64 videos, we identified instances in which two ants simultaneously gripped a stick with their mandibles and moved it together. The number of interactions per video ranged from 1 to 11, with a mean of 4.1 interactions per video. For analysis, we only included cases where the first ant was already moving the stick at the moment the second ant joined. For all cases of shared transport, three video frames were extracted: (1) the moment the first ant initially engaged with the stick, (2) the moment the second ant joined and (3) the moment the stick movement continued following the second ant's involvement. An ant was considered to have joined when it grasped the stick with its mandibles, adopted a stable body position and began to pull. The angle tool in ImageJ was used to measure all respective angles between the two ants, stick and stick movements from the extracted frames. We identified 132 first ants with 182 second ants joining these first ants. Additionally, we recorded the time elapsed between an ant's initial antennal contact with the stick and the moment it grasped the stick with its mandibles. If the stick movement remained within the video frame, we also measured the duration between the second ant joining and the moment it released and departed from the stick. More specifically, perseverance was measured for second ants that were aligned (<50 deg) or non-aligned (>130 deg) to the stick's direction of travel immediately prior the joining event (aligned: *n*=102 ants; non-aligned: *n*=58 ants) or the stick's direction of travel after the ant began to pull (aligned: *n*=85 ants; non-aligned: *n*=34 ants).

To further investigate grip adjustment behaviour, we selected instances of shared transport in which ants were either aligned (*n*=30 ants) or non-aligned (*n*=23 ants). Aligned was defined as cases where the second ant joined the stick at an angle of less than 50 deg relative to the first ant and the stick's direction of movement prior to joining. In contrast, non-aligned was defined by joining angles greater than 130 deg. To assess body posture adjustments and their potential impact, only cases in which the second ant remained engaged with the stick for at least 30 s were included. Within this 30 s period, the number of grip adjustments made by the second ant was recorded, and the angular magnitude of each adjustment was measured using ImageJ. A grip adjustment was defined as an ant releasing the stick from its mandibles and subsequently re-gripping the stick.

To assess inter-observer reliability, a second observer independently repeated 12% of the measurements of the angles for the second ant. The resulting mean angular differences were 5.2 deg for body orientations and 8.9 deg for travel directions.

### Statistical analysis and hypothesis

The Kolmogorov–Smirnov test was used to assess whether the angular distributions were uniformly distributed. Bimodality was evaluated using Hartigan's dip test, and the chi-square goodness-of-fit test was used to test for directional bias. The Wilcoxon rank-sum test was applied to compare the number and angular size of grip adjustments. We calculated empirical cumulative distribution functions using MATLAB's built-in survivor function for the time taken to grab a stick and for perseverance, and used a Cox proportional hazards model to analyse the time differences between groups.

Throughout the analysis, our overarching hypothesis was that ants joining a moving stick modify their behaviour in ways that facilitate collaborative transport. The null hypothesis was: there is no difference in the behaviour of ants when transporting nest material individually versus cooperatively, i.e. joining ants do not modify their behaviour in ways that facilitate the movement of the stick.

## RESULTS AND DISCUSSION

### Individual ants pull sticks and closely align their bodies to the stick

**Fig. 2.**
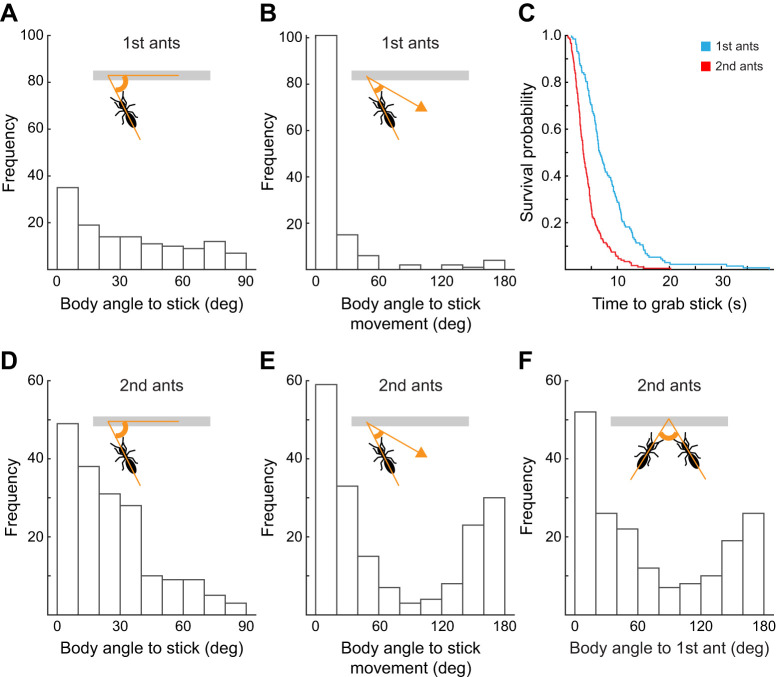
**Body orientation and behaviour of first and second ants during stick transport.** (A,B) Body angles of first ants (*n*=131) relative to the stick (A) and to the direction of stick movement (B) while moving the stick alone. Both distributions were significantly non-uniform, with ants aligning closely with the stick and pulling it toward themselves. (C) Survival curves illustrating the time from initial contact to first gripping the stick for ants encountering a stationary stick (first ants, *n*=132) versus those joining a moving stick (second ants, *n*=174). Second ants gripped more quickly than first ants. (D–F) Body angles of second ants (*n*=182) relative to the stick (D), to stick movement (E) and to the first ant's orientation (F). Second ants showed a strong alignment with the stick (D). Distributions in E and F were significantly bimodal, but ants were more likely to be aligned than non-aligned. For statistics, see Results.

To assess whether ants adopt specific body orientations during stick handling, and how this relates to stick movement, we measured the body angles of ants individually moving a stick, relative to both the stick itself and its subsequent travel direction. Body orientations relative to the stick were significantly non-uniform (Fig. 2A; *n*=131; one-sample Kolmogorov–Smirnov test, *P*<0.001), with individuals showing a strong preference for aligning nearly parallel to the stick's axis, i.e. favouring angles closer to 0 deg than to 90 deg (Hartigans' dip test, *P*=0.639; Chi-square goodness-of-fit test, *P*<0.001). Similarly, body angles relative to the stick's movement direction were significantly different from a uniform distribution (Fig. 2B; *n*=131; one-sample Kolmogorov–Smirnov test, *P*<0.001), with most ants pulling the stick toward themselves rather than pushing it forward (Hartigans' dip test, *P*=0.567; Chi-square goodness-of-fit test, *P*<0.001).

### Second ants show coordinated behaviour when joining a moving stick

To investigate ants' potential understanding of stick movement and cooperation, we examined whether ants joining an already moving stick exhibited ‘smart’ choices or simply chance-level behaviour. Therefore, we compared the behaviour of ‘second’ ants, aka joiners, with that of ‘first’ ants, the initiators of stick movement. Initiator ants grasping a stationary stick (*n*=132) exhibited significantly longer delays between initial contact and first gripping compared with second ants joining a moving stick [*n*=174; Fig. 2C; Cox proportional hazards model, HR=2.534, 95% confidence interval (CI): [1.997, 3.215], *P*<0.001], suggesting that a moving stick is more attractive or salient. It remains to be tested whether second-ant recruitment relies on an increased saliency of the stick, perhaps through motion cues or altered positions, or some form of explicit communication signal from first ants. Like first ants grasping a stationary stick, second ants joining a moving stick aligned nearly parallel to the stick (Fig. 2D; *n*=182; one-sample Kolmogorov–Smirnov test, *P*<0.001; Hartigans' dip test, *P*=0.860; Chi-square goodness-of-fit test, *P*<0.001). However, the distributions of joining angles differed significantly between first and second ants (two-sample Kolmogorov–Smirnov test, *P*=0.013), with second ants showing tighter alignment. To explore whether joining ants can perceive which way to grab the stick efficiently, we looked at the distribution of joining angles relative to the stick's direction of travel, which for second ants was significantly non-random (Fig. 2E; *n*=182; one-sample Kolmogorov–Smirnov test, *P*<0.001) and bimodal (Hartigans' dip test, *P*=0.001), with peaks near 0 deg and 180 deg, indicating alignment or anti-alignment respectively. Despite the presence of opposing orientations, ants were more likely to align than non-align (Chi-square goodness-of-fit test, *P*<0.001). As expected, a similar bimodal pattern was found in the angular differences between the body axis of two ants jointly carrying a stick (Fig. 2F; *n*=182; one-sample Kolmogorov–Smirnov test, *P*<0.001; Hartigans' dip test, *P*=0.001), again with a higher frequency of second ants aligning their body orientation with that of the first ant (Chi-square goodness-of-fit test, *P*<0.001).

While cooperative transport has been extensively studied in situations where workers share a clear directional goal, typically moving food towards the nest (Czaczkes et al., 2011; Franks, 1986; Franks et al., 2001; Moffett, 1988; Robson and Traniello, 1998), much less is known about cooperation when individuals lack a shared goal direction, as in nest maintenance. Early observations by Chauvin (1958, 1959a,b) showed that twigs placed on nest surfaces were rapidly incorporated into the mound through continuous turnover. For example, twig arrangements placed on the nest disappeared within minutes as workers dismantled and redistributed them (Chauvin, 1959b). The sticks used in our study were therefore expected to be integrated quickly. Wood ants seem to adjust their body posture and grip based on local sensory input (e.g. tactile, proprioceptive or visual motion cues) that gives feedback on success. The bimodal alignment pattern observed in joining ants, skewed toward cooperative positions, suggests that ants detect motion direction and modulate their behaviour accordingly, or that there is a directional asymmetry in an already moving stick in terms of which joining positions second ants can and want to join.

### Cooperative transport is promoted by behavioural heuristics

**Fig. 3.**
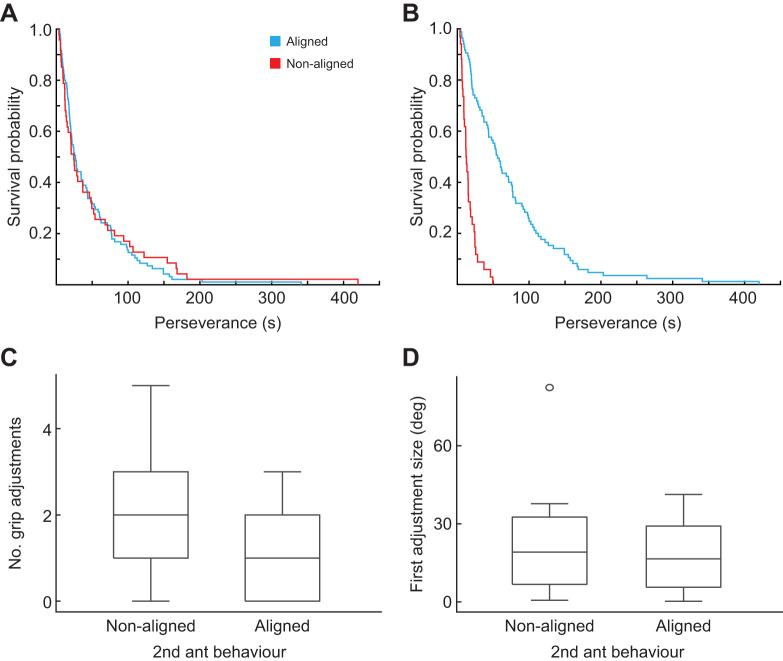
**Heuristic behavioural characteristics during cooperative transport.** (A,B) Survival curves illustrating perseverance (duration of stick contact) in second ants joining at misaligned (>130 deg, non-aligned; A: *n*=58; B: *n*=34) or aligned angles (<50 deg, aligned; A: *n*=102; B: *n*=85) relative to stick movement immediately prior to the joining event (A) or to the stick's direction of travel after the ant began to pull (B). Perseverance was significantly higher for aligned ants in B but not in A. (C) Number of grip adjustments for non-aligned (*n*=30) versus aligned second ants (*n*=23); aligned ants made significantly fewer adjustments. (D) Grip angle adjustment size for non-aligned (*n*=28) versus aligned second ants (*n*=14); grip angle did not differ between conditions. Boxplots show medians, upper and lower quartiles and interquartile ranges; circles are outliers. For statistics, see Results.

We examined perseverance and grip adjustment to test whether second ants use behavioural rules that promote cooperation. Perseverance, measured as engagement time, did not differ between aligned (*n*=102) and non-aligned (*n*=58) ants when assessed by the second ant's body angle relative to the stick's movement direction prior to joining (Fig. 3A; Cox proportional hazards model, HR=0.949, 95% CI: [0.665, 1.353], *P*=0.771). However, when perseverance was analysed relative to the stick's movement after the second ant joined, aligned ants (*n*=85) exhibited significantly longer engagement times than non-aligned ants (*n*=34; Fig. 3B; Cox proportional hazards model, HR=6.458, 95% CI: [3.922, 10.632], *P*<0.001).

Additional behavioural heuristics that promote cooperation become evident when examining the probability of grip adjustment. Second ants that were non-aligned with the first ant (*n*=30) performed a higher number of grip adjustments within the first 30 s of engagement compared with second ants that were aligned (*n*=23; Fig. 3C; Wilcoxon rank sum test, *P*<0.001), although the angular magnitude of these adjustments did not differ between groups (non-aligned: *n*=28; aligned: *n*=14; Fig. 3D; Wilcoxon rank sum test, *P*=0.622).

Ants that failed to align effectively made more frequent grip adjustments and disengaged earlier, consistent with mechanisms allowing individuals to evaluate task efficacy and end unhelpful behaviour (Dreyer et al., 2025; Feinerman et al., 2018; McCreery and Breed, 2014). The ants' ability to flexibly adjust their grip, posture and engagement time reveals behavioural heuristics that act as simple decision rules that are modulated by sensory input from direct interactions with the object.

### Sensorimotor heuristics underpin emergent cooperation

Cooperative transport relies on local control mechanisms, utilising a form of implicit communication transmitted through dynamic mechanical forces in the carried object (Czaczkes and Ratnieks, 2011; Dreyer et al., 2025; Feinerman et al., 2018; Gelblum et al., 2015). This might give ants local information to determine whether a current strategy is successful – information that can tune perseverance. However, theoretical models and behavioural experiments investigating collaborative food transport have suggested that cooperative transport is most effective when individuals are persistent (McCreery, 2017; McCreery et al., 2016), even if this can lead to deadlocks. In our study, ants modulated their persistence and grip so did not blindly continue unsuccessful attempts, but the rules of cooperation might be different for transport of nest material as compared with food transport. Food might be considered a higher value item, and retrieving food items means that cooperating ants have similar estimates of the direction back to the nest (Collett et al., 2025).

The contribution of ants implementing local rules as part of self-organising construction is established. Termites rely on olfactory-mediated feedback to guide placement of nest material (Bruinsma, 1979), leading to the coordinated building of galleries or chambers (Bonabeau et al., 1998; Stuart, 1967). Similarly, in *Lasius niger* ants, the construction of evenly spaced pillars is regulated through a comparable stigmergic mechanism (Khuong et al., 2016; Sudd, 1967). In both cases, complex architecture emerges from individuals following simple, local behavioural rules. However, there is some evidence that individual skills and experience also contribute. Termites have been observed selecting specific soil types for distinct structures, potentially indicating learned preferences (Jouquet et al., 2002). Similarly, *Temnothorax* ants show individual specialisation in handling different grain sizes, suggesting that workers refine their efficiency through experience (Aleksiev et al., 2007). It remains to be seen whether wood ants show plasticity and skill in the handling of nest material. It is possible that specialised workers show such skills, and that individuals learn from experience (Sugasawa et al., 2021), adapting heuristics to increase efficiency.

In conclusion, cooperative behaviour during nest maintenance can arise through simple behavioural rules without central coordination. This study highlights how sensorimotor heuristics driven by motion detection and local mechanical feedback can underpin emergent cooperation in environments where constant material rearrangement is required, as in wood ant nest maintenance (Frouz and Jilkova, 2008; Goryunov, 2015).
